# Burden of bloodstream infection in older persons: a population‐based study

**DOI:** 10.1186/s12877-020-01984-z

**Published:** 2021-01-07

**Authors:** Kevin B. Laupland, Kelsey Pasquill, Lisa Steele, Elizabeth C Parfitt

**Affiliations:** 1 Department of Intensive Care Services, Royal Brisbane and Women’s Hospital, Level 3 Ned Hanlon Building, Butterfield Street, 4029 Brisbane, Queensland Australia; 2 Faculty of Health, Queensland University of Technology (QUT), Brisbane, Queensland Australia; 3 Department of Medicine, Royal Inland Hospital, Kamloops, British Columbia Canada; 4 Department of Pathology and Laboratory Medicine, Royal Inland Hospital, Kamloops, British Columbia Canada

**Keywords:** Bloodstream infection, BSI, Adult, Population‐based study, Incidence

## Abstract

**Background:**

Advancing age is a major risk factor for developing and dying from bloodstream infections (BSI). However, there is a paucity of population-based studies investigating the epidemiology of BSI in older persons.

**Objective:**

To define the incidence, clinical determinants, and risk factors for death among those aged 65 years and older with BSI.

**Methods:**

Population-based surveillance was conducted in the western interior of British Columbia, Canada, between April 1, 2010 and March 31, 2020. Chart reviews were conducted for clinical details and all cause case-fatality was established at 30-days follow-up.

**Results:**

A total of 1854 incident BSI were identified among 1657 individuals aged 65 and older for an annual incidence of 533.9 per 100,000 population; the incidence for those aged 65-74, 75-84, and ≥85 years was 375.3, 678.9, and 1046.6 per 100,000 population, respectively. Males were at significantly increased risk as compared to females (incidence rate ratio, IRR 1.44; 95% confidence interval, CI, 1.32-1.59; *p*<0.0001). The crude annual incidence increased by 50% during the study. However, this was related to shift in population demographics with no increase evident following age- and sex-standardization. Older patients were more likely to have healthcare-associated infections and genitourinary sources and less likely to have bone/joint or soft tissue infections. The proportion of patients with underlying congestive heart failure, stroke, and dementia increased, whereas diabetes and liver disease decreased with older age. The overall 30-day all cause case-fatality rate was 22.0% (364/1657). After adjustment for clinical focus, onset of infection, etiology, and co-morbidity in a logistic model, those aged 75-84 years (odds ratio, OR, 1.66; 95% CI, 1.25-2.21) and ≥ 85 years (OR, 1.98; 95% CI, 1.41-2.77) were at significantly increased risk for death as compared to those aged 65-74 years.

**Conclusion:**

Bloodstream infection is common in older persons and is a major cause of death. Countries with aging populations worldwide should expect an increase burden associated with BSI in the coming years.

## Background

Older people represent the highest demographic risk group for morbidity and mortality from bloodstream infections (BSI) [1–3]. Although the underlying reason for excess risk among older persons has not been fully elucidated, the high rates of co-morbid illness, functional debility, and healthcare exposure experienced by this demographic are recognized as major determinants [4, 5]. A number of studies have shown that as compared to younger cohorts, older people have increased risks for Gram-negative infections, urinary source, and antimicrobial resistance [6–8]. The development of BSI is a serious condition with approximately one-quarter of older persons dying within a month of diagnosis [8].

Population-based studies are considered optimal to define the epidemiology of an infectious disease [9]. In these designs all cases occurring among residents of a defined geographical area are included, and as a result important selection biases are minimized [10]. However, most studies investigating BSI in older people are either hospital-based or focussed on highly selected cohorts [4, 6–8, 11, 12]. The objective of this study was to determine the contemporary incidence, evaluate secular changes, and establish the determinants and outcomes of BSI in a population of older Canadians.

## Methods

### Study population

Population-based surveillance for all BSI occurring among residents of the western interior of British Columbia, Canada aged ≥ 65 years was conducted [13]. The western interior region (total population ≈ 191,000) encompasses a large and geographically diverse area within the southern interior of the province of British Columbia. The Interior Health Research Ethics Board granted a waiver of individual informed consent (201314052-I).

### Study protocol

Incident BSI occurring among area residents between April 1, 2010 and March 31, 2020 were identified at the regional microbiology laboratory at Royal Inland Hospital in Kamloops. Prior to 2016–2017, this laboratory was the sole service provider for blood cultures in the region. Thereafter, a private laboratory started offering blood culture services for some outpatient collection sites and were not included in this study. These are estimated to represent  < 1% of all significant bloodstream isolates in the region. Non-residents based on postal code of primary address were excluded. Once potential cases were identified, previously established and validated algorithms were applied to confirm incident cases of disease and to classify episodes [14]. Repeat isolations of the same species within 30 days were deemed to represent the same episode of disease. Isolation of more than one species within a 48-hour period were classified as poly-microbial infections. Organisms commonly associated with contamination were required to have at least 2 sets positive within 5 days to be considered further as possible incident infections.

Once putative incident infections were identified, a senior infectious diseases consultant performed case-by-case electronic health record review in order to confirm the presence of an incident infection, establish the most likely focus of infection, and abstract clinical details. Comorbidities were recorded as per Charlson et al. [15], and episodes were classified as community-associated, healthcare-associated, and hospital-onset as per Friedman et al. [16]. Death at 30-days post index infection was determined using provincial vital statistics information [17].

### Analysis

Analysis was performed using Stata (version 15.1; StataCorp LP, College Station, TX, USA). Incidence rates were calculated using the annual population of the region available from the provincial registry [18]. We *a priori* chose to examine age groups categorized as 65–74, 75–84, and 85 years and older [7]. Study years were grouped to include the period from April 1 through to March 31 of the subsequent year (i.e. 2010 includes cases from April 1, 2010 through March 31, 2011). Crude incidence rates were calculated by the number of incident cases divided by the population aged ≥ 65 years and expressed as a rate per 100,000. Where subgroups were compared these were expressed as incidence rate ratios (IRR) with exact 95% confidence intervals (CI). To adjust for demographic population changes, annual incidence rates were sex- and age-adjusted to the 2019 population using direct methods and 5-year age strata.

Fisher’s exact test was used for comparison of group categorical data. Histograms were used to assess the underlying distribution of continuous data prior to analysis. Skewed variables were reported as medians with interquartile range (IQR). The median test was used to compare non-normally distributed continuous data across multiple groups. A logistic regression model was developed to examine independent risks for all cause 30-day case-fatality associated with BSI. In this analysis, only first episodes of BSI were included. Variables for inclusion in the initial model were *a priori* specified and included age group, sex, infection focus, etiologic group, Charlson comorbidity index, and location of onset of infection. Backward stepwise variable elimination and regrouping of variables was performed to develop the most parsimonious model. Final model calibration and discrimination were assessed using the Hosmer-Lemeshow and goodness of fit tests, respectively. *P*-values less than 0.05 were regarded as statistically significant for all analyses and adjustment for multiple testing was not performed.

## Results

During the decade of surveillance, 1854 incident infections were identified among 1657 western interior residents aged ≥ 65 years. One hundred and forty eight (8.0%) patients had second episodes and 35 (1.9%) had three, 11 (0.6%) had four, and three (0.2%) had five episodes of incident BSI. Overall, 318 (17.2%), 971 (52.4%), and 565 (30.5%) incident episodes were hospital-onset, healthcare-associated, and community-associated, respectively.

**Fig. 1:**
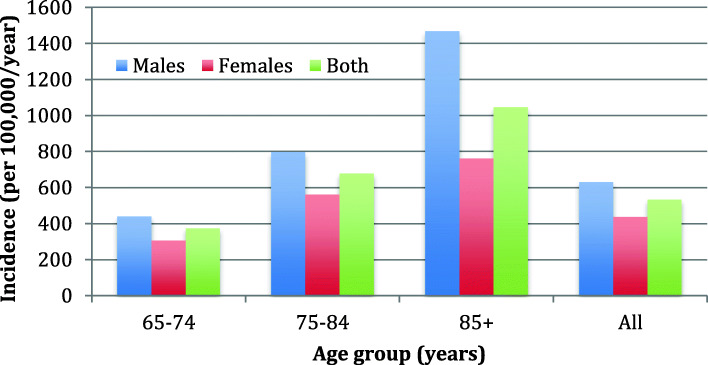
Incidence of bloodstream by age and sex, western interior 2010–2019

The overall crude annual incidence was 533.9 per 100,000 population, and was 375.3, 678.9, and 1046.6 per 100,000 for ages 65–74, 75–84, and 85 and older, respectively. Males were at significantly increased risk as compared to females (IRR 1.44; 95% CI, 1.32–1.59; *p* < 0.0001), and this difference was most pronounced in the oldest patients as shown in Fig. 1.

**Fig. 2:**
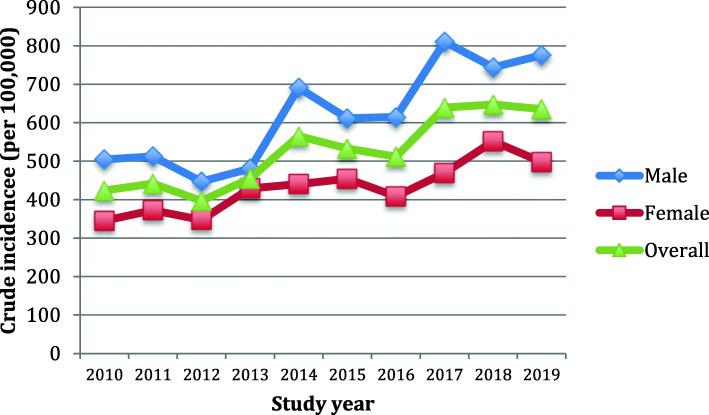
Crude incidence rates of bloodstream infection during a decade of surveillance

**Fig. 3:**
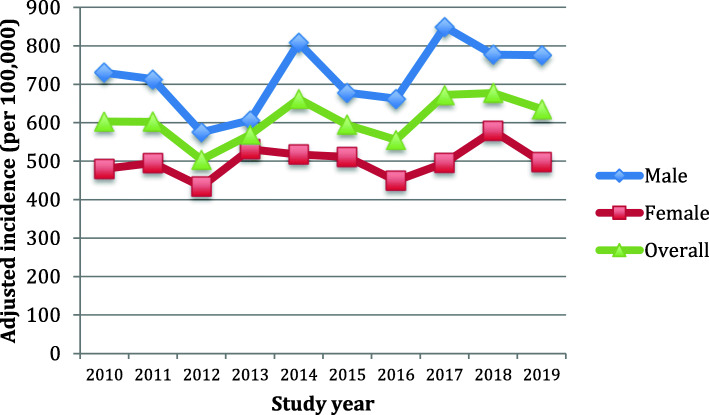
Sex- and age-adjusted incidence rates of bloodstream infection during a decade of surveillance

The crude incidence demonstrated both year-to-year variability and a progressive increase from the first (423.7 per 100,000) through last (635.3 per 100,000) years of study as shown in Fig. 2. However, following age- and sex-standardization, no evident linear trend for a change in annual incidence was observed during the study as shown in Fig. 3. The overall age- and sex-adjusted incidence was 607.5 per 100,000 annually.

Clinical determinants among those aged 65–74, 75–84, and 85 years and older are shown in Table 1. A decreasing proportion of community-onset BSI was observed with advancing age with a commensurate increase in healthcare-associated disease (Table 1). No significant difference was observed in the median Charlson Comorbidity Index score across the three age categories. However, when individual co-morbidities were assessed, congestive heart failure, cerebrovascular accident, and dementia increased with age, whereas diabetes and liver disease significantly decreased with older age (Table 1). Although no overall significant differences were observed in the distribution of BSI etiology by age group, those over age 85 years were less likely to have Gram-positive (114/370; 30.8% vs. 569/1484; 38.3%; *p* = 0.008) and more likely to have Gram-negative infections (213/370; 57.7% vs. 735/1484; 49.5%; *p* = 0.006). The most frequently isolated species included *Escherichia coli* (663; 35.8%), *Staphylococcus aureus* (277; 14.9%), *Klebsiella pneumoniae* (107; 5.8%), *Enterococcus faecalis* (86; 4.6%), *Streptococcus pneumoniae* (69; 3.7%), and *Pseudomonas aeruginosa* (57; 3.1%). Bone and joint and soft tissue infections decreased and genitourinary sources increased with advancing age (Table 1).

**Table 1:** Clinical determinants and outcome of bloodstream infections in older persons

Parameter	Age 65–74 *N* = 783	75–84 *n* = 701	84+ *n* = 370	*P*-value
Male	470 (60.3%)	410 (50.5%)	209 (56.5%)	0.5
Hospital-onset Healthcare-associated Community-associated	139 (17.8%) 377 (48.2%) 267 (34.1%)	121 (17.3%) 374 (53.4%) 206 (29.4%)	58 (15.7%) 220 (59.5%) 92 (24.9%)	0.006
Median (interquartile range) Charlson Comorbidity Index	2 (1–4)	2 (1–4)	2 (1–3)	0.9
Co-morbidity Myocardial infarction Congestive heart failure Peripheral vascular disease Stroke Plegia Chronic lung disease Diabetes mellitus Renal disease Liver disease Cancer Dementia Rheumatic disease HIV/AIDS	88 (11.2%) 65 (8.3%) 76 (9.7%) 59 (7.5%) 26 (3.3%) 163 (20.8%) 239 (30.5%) 90 (11.5%) 73 (9.3%) 229 (29.3%) 29 (3.7%) 58 (6.1%) 1 (0.1%)	87 (12.4%) 113 (16.1%) 67 (9.6%) 79 (11.3%) 18 (2.6%) 152 (21.7%) 213 (30.4%) 95 (13.6%) 21 (3.0%) 209 (29.8%) 104 (14.8%) 31 (4.4%) 0	54 (14.6%) 85 (23.0%) 26 (7.0%) 53 (14.3%) 13 (3.5%) 82 (22.2%) 74 (20.0%) 57 (15.4%) 4 (1.1%) 98 (26.5%) 95 (25.7%) 17 (4.6%) 0	0.3 < 0.001 0.3 0.001 0.6 0.8 < 0.001 0.2 < 0.001 0.5 < 0.001 0.3 1
Microbiology group Gram-positive Gram-negative Anaerobes Yeast Polymicrobial	308 (39.3%) 376 (48.0%) 33 (4.2%) 12 (1.5%) 54 (6.9%)	261 (37.2%) 359 (51.2%) 26 (3.7%) 5 (0.7%) 50 (7.1%)	114 (30.8%) 213 (57.6%) 15 (4.1%) 2 (0.54%) 26 (7.0%)	0.1
Clinical focus No focus Bone and joint Soft tissue Respiratory Cardiovascular Abdomen/pelvic Central nervous Genitourinary	121 (15.5%) 60 (7.7%) 76 (9.7) 84 (10.7%) 49 (6.3%) 161 (20.6%) 3 (0.4%) 229 (29.3%)	107 (15.3%) 37 (5.3%) 54 (7.7%) 74 (10.6%) 45 (6.4%) 147 (21.0%) 1 (0.1%) 236 (33.7%)	51 (13.8%) 14 (3.8%) 18 (4.9%) 48 (13.0%) 26 (7.0%) 68 (18.4%) 0 145 (39.2%)	0.016
Admitted to hospital	717 (91.6%)	645 (92.0%)	347 (93.8%)	0.4
Median length of stay in days (IQR) All patients Survivors to discharge	10 (5–24) 9 (5–21)	9 (4–19) 9 (5–19)	8 (5–18) 9 (5–20)	0.1 0.9
Day 30 case-fatality	138 (17.6%)	167 (23.8%)	101 (27.3%)	< 0.001

Among 1657 first episodes of BSI, day 30 all cause case-fatality was 17.3% (121/701), 24.6% (155/629), and 26.9% (88/327) for the ages 65–74, 75–84, and 85 and older, respectively. A multivariable logistic regression model was developed (*n* = 1657; area under receiver operator characteristic = 0.743; goodness of fit *p* = 0.6) and the results are displayed in Table 2.

**Table 2:** Logistic regression modeling of risk factors associated with death among 1657 first episodes of bloodstream infection

Variable	Odds Ratio (95% confidence interval)	*P*-value
Age group (years) 65–74 75–84 85 and older	1 (reference) 1.66 (1.25–2.21) 1.98 (1.41–2.77)	0.001 < 0.001
Onset category Hospital-onset Healthcare-associated Community-associated	1 (reference) 0.47 (0.35–0.64) 0.24 (0.16–0.35)	< 0.001 < 0.001
Etiology Other Yeast Polymicrobial	1 (reference) 2.79 (0.92–8.46) 1.75 (1.12–2.73)	0.07 0.014
Charlson comorbidity index (per point)	1.15 (1.08–1.21)	< 0.001
Infection focus Other Bone and joint Respiratory Genitourinary	1 (reference) 0.52 (0.29–0.94) 2.10 (1.47–2.99) 0.37 (0.27–0.52)	0.03 < 0.001 < 0.001

## Discussion

This study identifies the major burden associated with BSI among older persons. Based on an adjusted overall incidence of 607 per 100,000 population and 30-day case-fatality of 22%, the overall annual mortality rate in the present study was 133 per 100,000 population. The province of British Columbia has a population of approximately one million residents aged ≥ 65 years such that an estimated ≈ 1300 older persons in the province may be expected to die in 2020 as a result of BSI. In comparison, annualizing pandemic epidemiology data from January 15 through October 17, 2020 predicted an estimated ≈ 305 deaths due to COVID-19 among British Columbia residents aged 65 and older in 2020 [19]. While the potential for explosive increases in COVID-19 deaths cannot be underestimated, it is noteworthy that “baseline” BSI may be expected to result in a four-fold greater magnitude of suffering and death as compared to COVID-19 in its first pandemic year.

Although there are numerous studies that have focussed on selected cohorts, there are few population-based studies that have included all etiologies of BSI for which we may compare our results [3, 11, 20–23]. Notably, Crane et al. conducted retrospective surveillance for BSI among residents of Olmsted County, USA, during 2003–2005 with a focus on the location of onset of disease [11]. Among the 347 incident cases aged ≥ 65 years identified, 46%, 44%, and 10% were community-onset, healthcare-associated, and hospital-onset, and 14-day case-fatality rates were 6%, 15%, and 14%, respectively. We observed a slightly higher proportion of hospital onset cases and substantially higher overall case-fatality in our study. Mehl and colleagues examined incident BSI among all adult residents of mid-Norway during 2002–2013 and found a very high incidence in older persons, particularly among males with healthcare-associated disease [3]. Importantly, their reported overall incidence rates were 2–3 fold higher than those observed in our study. Like with our study, both the American and Norwegian studies found the urinary tract as the most important source of BSI in older persons.

Although there is a rich body of literature related to the epidemiology of BSI, older patients have infrequently been the focus [4–8]. This is somewhat surprising given that approximately one-half of all BSI occur in older persons and case fatality rates are highest in this cohort. A recent review of the published literature reported that the urinary tract is the most common source, that Gram-negative organisms are the most important agents of BSI in older persons, and that healthcare-associated disease is increased with age [8]. Our study findings are in congruence to this review. The influence of comorbid illness and advancing age *per se* on the risk for development and adverse outcome from BSI has been less well defined. Sogaard et al. studied age and comorbidity in relation to 30-day case-fatality among a population-based cohort of 2,851 patients (2001 age 65 and older) in North Denmark during 1995–2004 [4]. They found that both increasing co-morbidity and age were significantly associated with adverse outcome. It is noteworthy that we observed that some comorbidities (i.e. heart failure, stroke, and dementia) proportionally increased and others decreased (i.e. liver disease and diabetes) as shown in Table 1. This likely reflects in part the degree to which these co-morbidities may increase the risk for infection and the degree to which they result in death in association with advancing age.

There are some limitations of our study that merit discussion. First, collection of clinical information was retrospective such that our classification of cases was based on the information available in hospital records. We did however aim to minimize variability by conduct of all chart reviews by a single senior infectious diseases consultant. Second, as with all observational studies investigating BSI, ascertainment of cases is dependent on sampling of patients in order to identify those with positive blood cultures [24]. Although we have overall numbers of cultures processed by our laboratory, we do not specifically have data on which patients or age-groups are sampled. Third, we did not collect other variables that are potentially important contributors to outcome including time to adequate antimicrobial therapy and severity of illness including the presence of septic shock. Finally, our surveillance was not *a priori* designed to specifically examine BSI in older persons. As a result, we did not collect additional clinical measures of potential relevance including levels or degrees of comorbidities (i.e. severity of dementia), use of medical devices (i.e. indwelling urinary catheters), or functional capacities (i.e. activities of daily living) or degree of frailty.

## Conclusions

In summary, our study identifies the major burden of illness associated with BSI in older persons. We further identify the important role that advancing age plays on the determinants and outcome of BSI. The high incidence, morbidity, and mortality associated with BSI in older persons justifies prioritization of healthcare and research efforts to minimize its major burden. The importance of BSI as a cause of human suffering and death may be expected to increase as populations become increasing older and complicated by comorbid diseases.

## Data Availability

The datasets generated and/or analysed during the current study are not publicly available due institutional research ethics agreement but may be available from the corresponding author on reasonable request.

## Abbreviation

BSI: Bloodstream infection
